# Management of Patellar Chondral Defects with Autologous Matrix Induced Chondrogenesis (AMIC) Compared to Microfractures: A Four Years Follow-Up Clinical Trial

**DOI:** 10.3390/life11020141

**Published:** 2021-02-13

**Authors:** Filippo Migliorini, Jörg Eschweiler, Nicola Maffulli, Arne Driessen, Björn Rath, Markus Tingart, Hanno Schenker

**Affiliations:** 1 Department of Orthopedics and Trauma Surgery, University Clinic Aachen, RWTH Aachen University Clinic, 52064 Aachen, Germany; migliorini.md@gmail.com (F.M.); joeschweiler@ukaachen.de (J.E.); adriessen@ukaachen.de (A.D.); Bjoern.Rath@klinikum-wegr.at (B.R.); mtingart@ukaachen.de (M.T.); hschenker@ukaachen.de (H.S.); 2 Department of Medicine, Surgery and Dentistry, University of Salerno, Via S. Allende, 84081 Baronissi (SA), Italy; 3 School of Pharmacy and Bioengineering, Keele University School of Medicine, Thornburrow Drive, Stoke-on-Trent ST4 7QB, UK; 4 Centre for Sports and Exercise Medicine, Barts and the London School of Medicine and Dentistry, Queen Mary University of London, Mile End Hospital, 275 Bancroft Road, London E1 4DG, UK; 5 Department of Orthopedics, Klinikum Wels-Grieskirchen, A-4600 Wels, Austria

**Keywords:** knee, patella, autologous matrix induced chondrogenesis, AMIC, microfractures, chondral defects, management

## Abstract

Introduction: Evidence on the management of chondral defects of the patella arises from studies in which the patellofemoral joint was treated together with the femorotibial joint and primary and revision settings. Furthermore, the superiority of Autologous Matrix Induced Chondrogenesis (AMIC) over microfractures (MFx) for patellar chondral defects is uncertain. Therefore, the present study compared primary isolated AMIC versus MFx for focal unipolar chondral defects of the patellar facet joints at midterm follow-up. Methods: Patients undergoing AMIC or isolated MFx surgery for borderline-sized focal unipolar chondral defects of the patellar facet joints were followed at our institution. All surgeries were performed in the same fashion by experienced surgeons. A parapatellar arthrotomy was adopted in all surgeries. The outcomes of interest were: Visual Analogic Scale (VAS), Tegner Activity Scale, International Knee Documentation Committee (IKDC), and the Lysholm scores. The Magnetic Resonance Observation of Cartilage Repair Tissue (MOCART) was assessed by a blinded radiologist, who had not been involved in the clinical management of the patients. Results: 38 patients were enrolled in the present study: 27 underwent AMIC, and 11 MFx. The mean follow-up was 45.1 months. The mean age of the patients at baseline was 34.5 years. The mean size of the defect was 2.6 cm^2^. The MFx cohort experienced a shorter length of the hospitalization (*P* = 0.008). There was no difference in terms of follow-up and previous symptoms duration, mean age, sex, side, defect size, and BMI. At last follow-up, the AMIC cohort reported greater IKDC (*P* = 0.01), Lysholm (*P* = 0.009), and Tegner (*P* = 0.02), along with a low rate of failure (*P* = 0.02). VAS was lower in the AMIC group (*P* = 0.002). No difference was found in the MOCART score (*P* = 0.09), rates of revision (*P* = 0.06), and arthroplasty (*P* = 0.2). Conclusion: The AMIC procedure achieves greater IKDC and Lysholm score, and a significant reduction of the VAS score in the management of patellar chondral defects. The Tegner scale demonstrated greater activity after AMIC procedure. Finally, the AMIC group evidenced a lower rate of failure. Similarity was found on MOCART score, rates of revision, and arthroplasty between the two procedures.

## 1. Introduction

Symptomatic focal chondral defects of the patella are common [1,2]. If left untreated, they can lead to early osteoarthritis [3]. Several surgical approaches have been proposed to manage chondral defects [4,5,6,7,8,9]. Microfractures (MFx) is cost-effective and has been largely used to manage smaller chondral defects [4,5]. Autologous chondrocyte implantation (ACI) techniques evolved from the periosteal patch (pACI), to chondral patch (cACI), to third generation procedures named matrix-induced ACI (mACI) [2,10,11,12]. Irrespective of the generation, ACI requires harvesting, external chondrocyte expansion, and a second stage surgery for re-implantation [13,14,15]. The superiority of ACI compared to MFx is still controversial [16,17,18]. Autologous Matrix-Induced Chondrogenesis (AMIC) avoids cartilage harvest and external expansion and is performed in a single surgical session [19,20]. Differently to ACI, which requires expanded autologous chondrocytes, AMIC exploits the potential of bone marrow-derived mesenchymal stem cells [21,22]. Isolated AMIC for chondral defects of the patella joint surface in a primary setting has been poorly investigated. Evidence on the management of chondral defects of the patella mainly arises from studies in which the patellofemoral joint was treated together with the femorotibial joint, and primary and revision settings were mixed [23,24,25,26]. Especially for borderline sized defects, the superiority of AMIC with respect to MFx for patellar defects is uncertain. The present study compared primary isolated AMIC versus MFx for focal unipolar chondral defects of the patellar facet joints at midterm follow-up.

## 2. Material and Methods

### 2.1. Patients Recruitment

**Figure 1:**
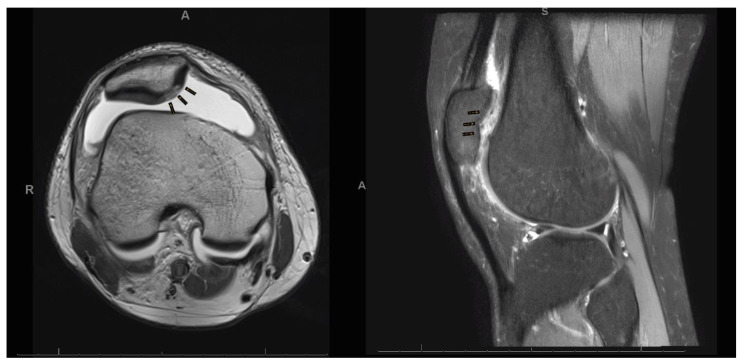
MRI of a focal chondral defect of the patella.

The present study was conducted according to the Consolidated Standards of Reporting Trials: the CONSORT statement [27]. This study was conducted in the Department of Orthopaedic Surgery of the RWTH University Hospital of Aachen between 2012 and 2020. All patients undergoing primary isolated AMIC or MFx for focal unipolar chondral defects of the patellar facet joints were consecutively enrolled. The inclusion criteria were: (1) symptomatic patellar chondral defect, (2) isolated focal defect sized 1.5 to 3.5 cm^2^, and (3) MRI evidence (Figure 1). The exclusion criteria were: (1) bilateral lesions, (2) multifocal lesions, (2) previous knee surgeries, (5) patellofemoral instability or history of dislocations, (6) lateral patellar compression syndrome, (7) any bone deformity, (8) radiographic evidence of osteoarthritis, and (7) any other relevant pathology that can influence the study. Suitable patients were asked to participate in this study pre-operatively. Patients were informed about pros and cons of both techniques, and they were free to choose between AMIC or MFx. In 2020, all patients were invited to participate in the present study, which was approved and registered by the ethics committee of the RWTH University of Aachen (project ID EK 438-20) and was performed according to the principles expressed in the Declaration of Helsinki. All patients were able to understand the nature of their treatment and provided written consent to use their clinical and imaging data for research purposes.

### 2.2. Surgical Technique

All the surgeries were performed in the same fashion by two experienced surgeons according to a previous report [28]. Briefly, preliminary arthroscopy was performed through traditional anteromedial and anterolateral portals. Debridement and curettage of the non-viable tissues surrounding the lesion were performed. MFx to a depth of 4 mm were performed using a 65° and 90° pick. In those patients who underwent AMIC, a mini medial parapatellar approach was performed. The perforation of the subchondral bone was performed with a 40° pick or a 1.2/1.4 mm Kischer wire under constant irrigation. An aluminum template was trimmed according to the defect. A type I/III porcine resorbable collagen membrane was used in all procedures (Chondro-Gide, Geistlich Pharma AG, Wolhusen, Switzerland). The membrane was trimmed according to the aluminum template to slightly undersize the defect to avoid displacement. The membrane was hydrated in a saline solution and placed into the lesion and attached with fibrin glue. The stability of the membrane was checked by repeatedly flexing and extending the knee. The rehabilitation process was performed according to our previous study [29].

### 2.3. Outcomes of Interest

On admission, the following data were recorded: age, gender, side, area of defect, BMI (Kg/m^2^), symptoms duration prior to surgery, and length of hospital stay. The primary outcomes of interest were to compare clinical findings using patient reported outcomes measures (PROMs) and complications of both groups. The secondary outcomes of interest were to compare MRI findings according to the Magnetic Resonance Observation of Cartilage Repair Tissue (MOCART) score [30]. At last follow-up, patients performed independently the following scores: Visual Analog Scale (VAS) [31], Tegner Activity Scale [32], International Knee Documentation Committee (IKDC) [33], and the Lysholm [34] scores. Data concerning the rate of complications (failure, revision, arthroplasty, delamination, hypertrophy) and additional procedures were also collected. Failure was defined as persistent pain that affected negatively the quality of life and limited the participation to recreational activities. The MOCART score was performed by a blinded radiologist, who had not been involved in the clinical management of the patients.

### 2.4. Statistical Analysis

All statistical analyses were performed using the software IBM SPSS version 25. Continuous data were analysed using the mean difference (MD), while dichotomic data with odd ratio (OR) effect measures for parametric data. The *T*-test and χ^2^ tests were performed, respectively, with values of *P* < 0.05 considered statistically significant. The confidence interval (CI) was set at 95% in all comparisons.

## 3. Results

### 3.1. Recruitment Process

**Figure 2:**
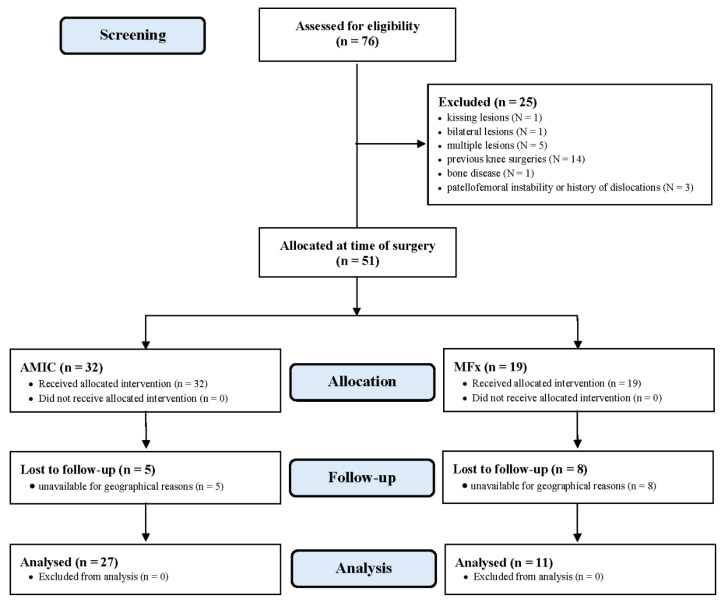
Diagram of the recruitment process.

A total of 76 patients were initially screened. Of them, 25 were not eligible: kissing lesions (*N* = 1), bilateral lesions (*N* = 1), multiple lesions (*N* = 5), previous knee surgeries (*N* = 14), metabolic bone disease (*N* = 1), and patellofemoral instability or history of dislocations (*N* = 3). Fifty-one patients were available and operated: 32 underwent AMIC and 19 microfractures. At last follow-up, five patients who had undergone AMIC and eight microfractures were not available. Eventually, 38 patients were enrolled in the present study: 27 underwent AMIC, and 11 MFx. The diagram of the recruitment process is shown in Figure 2.

### 3.2. Patients Demographics

The mean length of the follow-up was 45.1 months. The mean age of the patients at baseline was 34.5 years. The mean size of the defect was 2.6 cm^2^ (1.1 to 3.0). Fifty percent (19 of 38 patients) were women, and in 61% (23 of 38) of patients the right side was affected. The MFx cohort evidenced a shorter length of hospitalization (*P* = 0.008). There was no difference in terms of follow-up and previous symptoms duration, mean age, sex, side, defect size, and BMI. Demographic data of the patients are shown in Table 1.

### 3.3. Outcomes of Interest

At last follow-up, the AMIC cohort reported greater IKDC (*P* = 0.01), Lysholm (*P* = 0.009), and Tegner (*P* = 0.02). VAS was lower in the AMIC group (*P* = 0.002). No difference was found in the MOCART score (*P* = 0.09). These results are shown in greater detail in Table 2.

### 3.4. Complication

The AMIC group reported a lower rate of failure (*P* = 0.02). No difference was found in the rates of revision (*P* = 0.06) and arthroplasty (*P* = 0.2). No delamination or hypertrophy were reported at the last follow-up. These results are shown in greater detail in Table 3.

## 4. Discussion

According to the main findings of the present study, for patellar chondral defects, AMIC achieves greater IKDC and Lysholm score, along with a significant reduction of the VAS score. The Tegner scale demonstrated greater sporting activity after AMIC. Finally, the patients in the AMIC group experienced lower rate of failure. Similarity was found on MOCART score, rates of revision, and arthroplasty between the two procedures.

Up to 60% of patients undergoing knee arthroscopy evidence chondral defect [35]. Of them, up to 37% are located in the patellar facets [36,37,38,39]. Previous studies concerning patients treated for patellar chondral defects with autologous chondrocyte implantation (ACI) reported acceptable clinical results [35,40,41,42,43,44,45,46,47]. However, in these studies the patellofemoral joint was mainly treated along with the femorotibial joint, and primary and revision settings were not considered separately [23,24,25,26]. Tradati et al. [48] recently published a retrospective survey including 14 patients who underwent primary AMIC for isolated patellar defects. At a mean of 68.2 months follow-up, 78% of patients rated their outcomes as excellent [48]. They evidenced a statistically significant increase of the Tegner, Kuajala, and IKDC scores [48]. We were unable to identify other studies which compared AMIC vs. MFx for primary isolated chondral defect of the patella. MFx is suggested for chondral defect sized up to 2.5 cm^2^, while AMIC has been proposed for bigger defects [5,19,20,21,22,49,50,51,52]. The present cohort of patients had a mean size of 2.6 cm^2^, which can be considered borderline. The choice between AMIC rather than MFx in these patients can be questionable. MFx requires shorter surgical duration, can be performed arthroscopically, and allows quicker recovery. These features make MFx of special interest, also in patients with borderline size area of the defect. However, this study demonstrated that AMIC can be preferred over MFx for patients with a mean defect size of 2.6 cm^2^. The resorbable membrane we employed in all surgeries (ChondroGide) promotes the formation of the blood clot, which is rich in bone marrow-derived mesenchymal stem cells [24]. The bilayer structure of the membrane allows migration and proliferation of cells on its internal porous side, providing protection from the knee cavity thought its external compact layer.

The patellofemoral joint is complex, with intricate architecture and biomechanics. Chondral defects of the patella are often secondary to malalignment and instability syndromes, which make their treatment very challenging [53,54]. In patients with patellar chondral defects, pathoanatomical tissue morphology predisposing to patellofemoral instability (PFI) must be investigated [55,56]. Chondral defects are common in patients who experienced a patellar dislocation, [57,58,59,60,61,62]. Up to 96% of patients after patellar dislocation experience chondral damages [63,64,65,66]. Surgeons must be aware of previous patellar dislocations or potential subluxations. Evaluation of possible varus or valgus deformity and of the result of the apprehension test are mandatory [67,68]. Trochlear dysplasia, Install-Salvati ratio ranging from 1.0 and 1.2, and tibial tubercle-trochlear groove (TT-TG) greater than 22 mm also have been evaluated [69,70]. Another cause of persistent pain and possible chondral damage is the lateral patellar compression syndrome (LPCS) [71,72]. LPCS presents shortened and tightened lateral retinacular combined with lateral patellar tilt, with overload of the lateral patellar articular compartment and patellar mal-tracking [73,74,75]. Isolated lateral retinacular release or lateral retinacular lengthening are indicated for LPCS [76,77,78,79,80,81,82], restoring patellar tracking, and preserving the surfaces from further degeneration [83,84]. Further, femoral anteversion, generalized ligament laxity, and patellar hypermotility may represent other underlying causes of chondral defect [85,86] and should also be evaluated before planning surgery. It is important during clinical examination to evaluate these aforementioned risk factors. Indeed, for these patients, it would be recommended to combine AMIC with proximal or a distal realignment procedure to address the underlying cause of chondral damage.

This study has several limitations. Patients were not randomised and not blinded to the treatment received. The limited number of the patients included in the present investigation may limit the ability to detect uncommon complications. The 13 patients who did not attend the last follow-up were contacted telephonically and declared themselves satisfied but unavailable to attend for assessment for geographical reasons. Studies involving larger population and longer follow-ups are required. In the present study, the histological features of cartilage regeneration were not evaluated and the morphological appearance was assessed qualitatively through the MOCART score. However, the quality of the morphological assessment of the MOCART score is controversial. Previous studies evidenced that its potential to predict clinical outcome is limited [87,88]. The strict eligibility criteria, along with the good comparability between the two groups are however important strengths of this study. Experienced surgeons performed the procedures in a standardized fashion, with no preference of one over the other technique. Postoperative cares and rehabilitation protocols were highly standardized, thus representing further important strengths of the present study.

## 5. Conclusions

AMIC procedure achieves greater IKDC and Lysholm score, along with a significant reduction of the VAS score as management of patellar chondral defects. The Tegner scale demonstrated greater activity after AMIC procedure. Finally, the AMIC group evidenced lower rate of failure. Similarity was found on MOCART score, rates of revision, and arthroplasty between AMIC and MFx.

## Figures and Tables

**Table 1:** Demographic data of the patients (n.s.: not significant).

Endpoint	AMIC (*n* = 27)	MFx (*n* = 11)	*P*
Follow-up (months)	45.1 ± 22.9	49.1 ± 33.7	n.s.
Mean age	35.8 ± 14.5	31.4 ± 11.3	n.s.
Sex (female)	48% (13 of 27)	55% (6 of 11)	n.s.
Side (leg)	59% (16 of 27)	58% (7 of 11)	n.s.
Side (joint)			
Medial facet	48% (13 of 27)	72% (8 of 27)	
Lateral facet	52% (14 of 27)	28% (3 of 27)	
Symptoms duration (months)	68.7 ± 78.4	71.4 ± 83.3	n.s.
Hospitalization (days)	2.8 ± 1.1	1.8 ± 0.7	0.008
Area of defect (cm^2^)	2.7 ± 1.6	2.5 ± 0.6	n.s.
BMI (kg/m^2^)	26.9 ± 5.2	25.1 ± 3.4	n.s.

**Table 2:** Main results at last follow-up.

Endpoint	AMIC (*n* = 27)	MFx (*n* = 11)	95% CI	MD	*P*
IKDC	89.3 ± 16.4	74.9 ± 11.5	3.375 to 25.425	14.4	0.01
Lysholm	89.6 ± 10.5	78.1 ± 14.0	3.100 to 19.900	11.5	0.009
MOCART	70.0 ± 17.3	58.8 ± 18.9	1.683 to 24.083	11.2	0.09
VAS (0–10)	1.3 ± 0.7	2.5 ± 1.5	1.918 to 0.482	1.2	0.002
Tegner	5.0 ± 1.7	3.5 ± 1.9	0.225 to 2.775	1.5	0.02

**Table 3:** Complications: rate of revision, arthroplasty, and failure.

Endpoint	AMIC (*n* = 27)	MFx (*n* = 11)	OR	95% CI	*P*
Revision	4% (1 of 27)	27% (3 of 11)	0.103	0.0093 to 1.1282	0.06
Arthroplasty	0% (0 of 27)	9% (1 of 11)	0.1273	0.0048 to 3.3780	0.2
Failure	4% (1 of 27)	36% (4 of 11)	0.067	0.0065 to 0.7022	0.02

## Data Availability

The data presented in this study are available in this article.
